# Effects of Blood Flow Restriction Training on Muscular Strength and Hypertrophy in Older Individuals: A Systematic Review and Meta-Analysis

**DOI:** 10.1007/s40279-018-0994-1

**Published:** 2018-10-10

**Authors:** Christoph Centner, Patrick Wiegel, Albert Gollhofer, Daniel König

**Affiliations:** 1 Department of Sport and Sport Science, University of Freiburg, Freiburg, Germany; 2 Bernstein Center Freiburg, University of Freiburg, Freiburg, Germany

## Abstract

**Background:**

The combination of low-load resistance training with blood flow restriction (BFR) has recently been shown to promote muscular adaptations in various populations. To date, however, evidence is sparse on how this training regimen influences muscle mass and strength in older adults.

**Purpose:**

The purpose of this systematic review and meta-analysis was to quantitatively identify the effects of low-load BFR (LL-BFR) training on muscle mass and strength in older individuals in comparison with conventional resistance training programmes. Additionally, the effectiveness of walking with and without BFR was assessed.

**Methods:**

A PRISMA-compliant systematic review and meta-analysis was conducted. The systematic literature research was performed in the following electronic databases from inception to 1 June 2018: PubMed, Web of Science, Scopus, CINAHL, SPORTDiscus and CENTRAL. Subsequently, a random-effects meta-analysis with inverse variance weighting was conducted.

**Results:**

A total of 2658 articles were screened, and 11 studies with a total population of *N* = 238 were included in the meta-analysis. Our results revealed that during both low-load training and walking, the addition of BFR elicits significantly greater improvements in muscular strength with pooled effect sizes (ES) of 2.16 (95% CI 1.61 to 2.70) and 3.09 (95% CI 2.04 to 4.14), respectively. Muscle mass was also increased when comparing walking with and without BFR [ES 1.82 (95% CI 1.32 to 2.32)]. In comparison with high-load training, LL-BFR promotes similar muscle hypertrophy [ES 0.21 (95% CI − 0.14 to 0.56)] but lower strength gains [ES − 0.42 (95% CI − 0.70 to − 0.14)].

**Conclusion:**

This systematic review and meta-analysis reveals that LL-BFR and walking with BFR is an effective interventional approach to stimulate muscle hypertrophy and strength gains in older populations. As BFR literature is still scarce with regard to potential moderator variables (e.g. sex, cuff pressure or training volume/frequency), further research is needed for strengthening the evidence for an effective application of LL-BFR training in older people.

**Electronic supplementary material:**

The online version of this article (10.1007/s40279-018-0994-1) contains supplementary material, which is available to authorized users.

## Key Points

**Table:** 

The results of the present systematic review and meta-analysis suggest that blood flow restriction (BFR) is an effective strategy for increasing the effects of low-load (LL) resistance training and walking on muscle mass and strength in older adults.
In comparison with high-load (HL) resistance training, LL-BFR training produces comparable changes in muscle mass but lower increases in muscular strength.
The addition of BFR to LL resistance training or walking is an effective exercise alternative for older populations, for whom a traditional HL training might be contraindicated due to comorbidities or high mechanical stress to bones and joints.

## Introduction

In recent years, blood flow restriction (BFR) training has gained increasing attention in the scientific community [1–3]. By applying tourniquets or inflatable cuffs at the proximal portion of the limb, low-load BFR (LL-BFR) training (20–30% one repetition maximum, 1RM) has been shown to promote muscular hypertrophy and strength increases comparable to what is typically seen following high-load (HL) training programmes with 70–85% 1RM [4–6]. The advantage of low loads and thus reduced mechanical stress for joints and bones [7] is of particular interest for populations who are not capable of lifting near-maximum loads or for whom high loads may be contraindicated, such as in clinical rehabilitation.

In this context, particularly in elderly subjects, HL resistance training is often not feasible due to comorbidities such as coronary heart diseases, diabetes mellitus or musculoskeletal impairments [8–10]. With advancing age, the skeletal muscle mass decreases by as much as 3–8% per decade after the age of 30 [11]. The coexistence of both, a decrease in muscle mass and strength is termed *sarcopenia* [12] and has major functional and metabolic consequences, including an increased risk of falls and mortality [13, 14]. With regard to demographic changes, especially in Western societies [15], it is increasingly important to identify suitable evidence-based interventions that counteract the functional decline occurring with progressive age.

To maximize the span of effective functioning with advancing age, exercise and nutritional interventions have been suggested as the cornerstones in the management of sarcopenia [12, 16]. In particular, the prescription of long-term HL resistance training programmes has been shown to maintain and increase both muscle mass and strength [17–20]. However, these training regimens do not consider the high prevalence of comorbidities [8] and the decreased tolerance of mechanical stress in older individuals.

Although recent reviews have investigated the effects of LL-BFR training in athletes [21] and individuals with a clinical musculoskeletal condition [2], there is currently no systematic review summarizing the effects of LL-BFR in older adults. Thus, the aim of the present systematic review and meta-analysis is to assess the effects of LL-BFR training on muscle strength and muscle mass in older subjects and provide practical implications for the prevention and treatment of the age-induced decline in muscle mass and strength.

## Methods

### Search Strategy

This systematic review and meta-analysis followed the guidelines provided in the PRISMA statement [22] (Prospero registration number: CRD42018089980). For identification of relevant studies, a systematic literature search was performed by two researchers (CC & PW). The following electronic databases were searched from inception to 1 June, 2018: PubMed, Web of Science, Scopus, CINAHL, SPORTDiscus and CENTRAL. The search string was created with two sections: the first encompassed synonyms for LL-BFR training while the second was composed of synonyms for the topic of aging. To ensure that at least one search term within one section was included in the results, all synonyms were connected with the operator ‘OR’ and both sections were connected with the operator ‘AND’. Moreover, truncations and adjacency searching were used to find variations of the corresponding term and to restrict the results to specific ordered terms. Database searching was performed with no restrictions (‘All field/All text’ search) except in Scopus where the search was restricted to ‘Title, Abstract, Keywords’.

The search was conducted independently by the two researchers using the following search string for all databases: “blood flow restriction” OR “occlusion training” OR “vascular occlusion” OR KAATSU OR “ischemi* training” AND old* OR elder* OR sarcopeni* OR “musc* atrophy”.

**Fig. 1:**
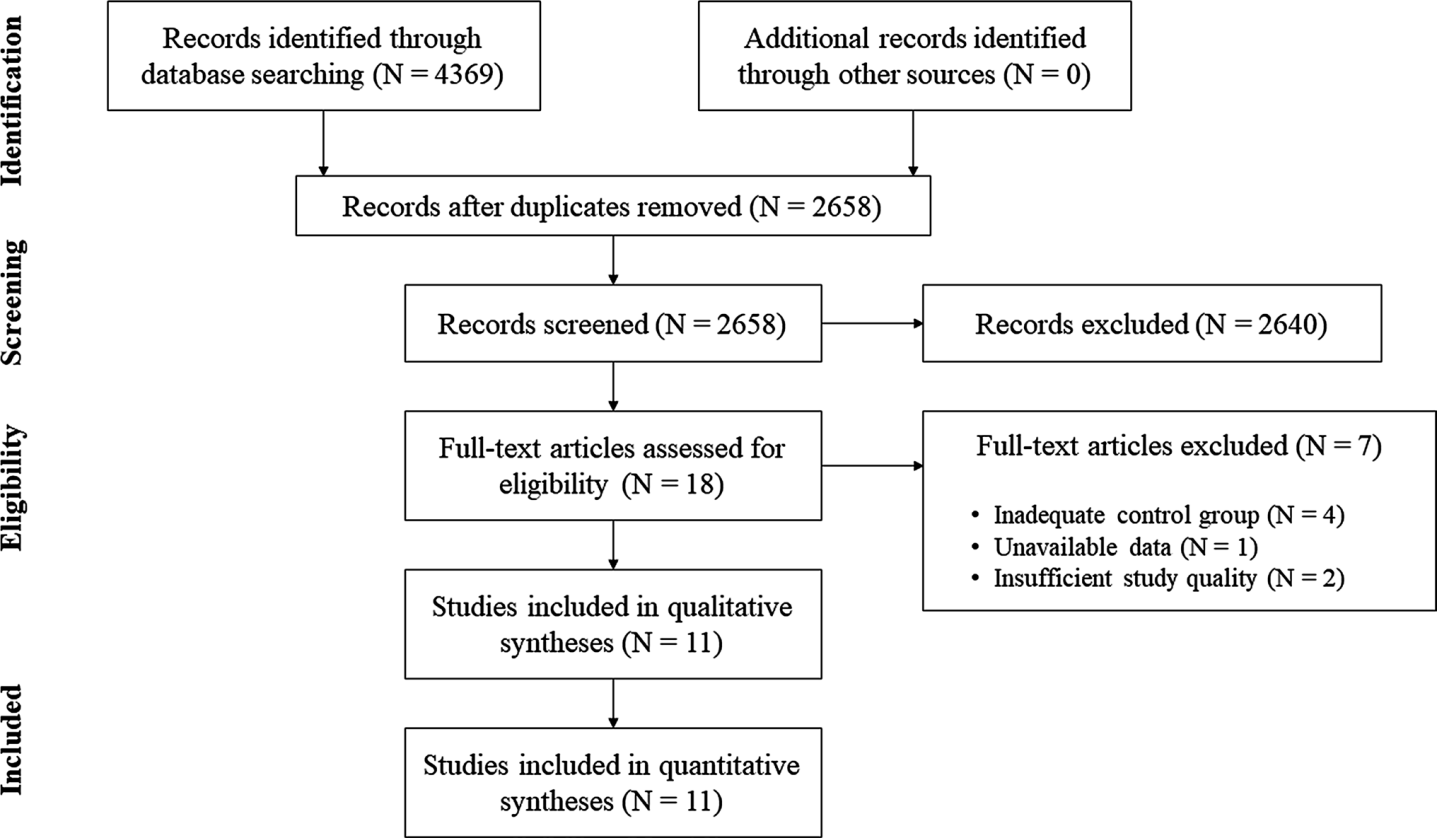
Flow chart presenting the search process and study selection

Study information, including title and abstract, were exported from the databases and stored in a citation manager. Before further processing of the studies all duplicates were removed (for search process see Fig. 1).

### Inclusion and Exclusion Criteria

All studies were screened and assessed for eligibility with regard to our inclusion and exclusion criteria, which were based on the PICOS principle (i.e. extracting population, intervention, comparison intervention, outcome measures and study design information) [23, 24]. Studies were considered relevant if (1) subjects were healthy older people (aged > 50 years), (2) the study design allowed comparisons between resistance training with and without vascular occlusion [HL (> 70% 1RM) or LL (< 50% 1RM) resistance training] or between walking with and without simultaneous BFR, (3) muscle mass and/or strength were assessed pre- and post-training.

Studies were not considered relevant if (1) participants had received a substance previously shown to result in muscle gains or (2) the manuscript was not written in the English language. Additionally, quality of reports was determined using the Physical Evidence Database (PEDro) scale, which is based on the Delphi list [25] (Electronic Supplementary Material Table S1). Studies with a score < 4 were excluded from this systematic review. For each of the 11 items of the PEDro scale, two reviewers (CC & PW) assessed the studies independently. In case of any discrepancy, a third reviewer (DK) evaluated the study to find a consensus.

### Data Extraction and Assessment of Reviewer Agreement

After screening of the studies, all relevant considered articles were assessed for eligibility based on their full texts. At this stage, we extracted information about (1) population characteristics, (2) primary outcome measures, (3) methods, (4) exercise/interventional characteristics and (5) the main result of the study. When intervention effects were assessed at multiple time points, only the very last time point was considered (as post-training value). In case of incomplete raw data availability, we contacted the corresponding author of the manuscript or extrapolated the data from figures, if the authors could not be reached. All studies were assessed for inclusion in this systematic review independently by two researchers (CC and PW) based on the extracted information. If there were any disagreements about inclusion of a study, a third reviewer (DK) was consulted. The extracted data of included studies are depicted in Tables 1, 2, 3 and 4.

**Table 1:** LL-BFR training and changes in muscle strength

Study	Subjects	Protocol	*N*	Exercise mode	Duration/frequency	Strength measurement	Percentage increase	Conclusion
Cook et al. [81]	Older adults (≥ 65 y)	LL-BFR (30–50% 1RM) HL (70% 1RM)	12 12	Leg curl Leg extension Leg press	12 wk; 2 days/wk	Isometric leg extension Dynamic leg curl Dynamic leg extension Dynamic leg press	LL-BFR: 10–26% HL: 18–56%	No significant between-group differences except for dynamic leg extension (greater in HL)
Karabulut et al. [4]	Older men (50–64 y)	LL-BFR (20% 1RM) HL (80% 1RM)	13 13	Leg press Leg extension	6 wk; 3 days/wk	Dynamic leg press Dynamic leg extension	LL-BFR: 19% HL: 20–31%	No significant between-group differences except for dynamic leg extension (greater in HL)
Libardi et al. [82]	Older adults (> 60 y)	LL-BFR (20–30% 1RM) HL (70-80% 1RM)	10 8	Leg press	12 wk; 2 days/wk	Dynamic leg press	LL-BFR: 35% HL: 38%	No significant between-group differences
Patterson and Ferguson [83]	Older adults (62–73 y)	LL-BFR (25% 1RM) LL (25% 1RM)	10 10	Plantar-flexion	4 wk; 3 days/wk	Isokinetic plantar flexion 0.52; 1.05; 2.09 rad/s Isometric plantar flexion Dynamic plantar flexion	LL-BFR: 11–20% LL: 0–4%	Greater strength improvements for LL-BFR except for isokinetic torque at 1.05 rad/s and 2.09 rad/s
Shimizu et al. [84]	Older adults (> 65 y)	LL-BFR (20% 1RM) LL (20% 1RM)	20 20	Leg extension Leg press Rowing Chest press	4 wk; 3 days/wk	Dynamic leg extension Dynamic leg press Dynamic rowing Dynamic chest press	LL-BFR: 6–19% LL: − 2 to 7%	No significant between-group differences
Thiebaud et al. [85]	Older women (61 ± 5 y)	LL-BFR (10–30% 1RM) HL (70–90% 1RM)	6 8	Seated chest press Seated row Seated shoulder press	8 wk; 3 days/wk	Dynamic chest press Dynamic row Dynamic shoulder press	LL-BFR: 4–10% HL: 5–18%	No significant between-group differences
Vechin et al. [86]	Older adults (59–71 y)	LL-BFR (20–30% 1RM) HL (70–80% 1RM)	8 8	Leg press	12 wk; 2 days/wk	Dynamic leg press	LL-BFR: 17% HL: 54%	HL tends to result in greater strength increases
Yasuda et al. [33]	Older women (61–86 y)	LL-BFR (35–45% 1RM) HL (70–90% 1RM)	10 10	Squats Knee extension	12 wk; 2 days/wk	Isometric knee extension Isometric knee flexion Dynamic leg press Dynamic knee extension	LL-BFR: 7–17% HL: 4–18%	Significant increase in LL-BFR group, no significant increase in HL group

*1RM* one-repetition maximum, *CON* control group, *HL* high-load, *LL* low-load, *LL-BFR* low-load blood flow restriction, *wk* week/s, *y* years

**Table 2:** LL-BFR training and changes in muscle mass

Study	Subjects	Protocol	*N*	Exercise mode	Duration/frequency	Muscle mass assessment	Percentage increase	Conclusion
Cook et al. [81]	Older adults (≥ 65 y)	LL-BFR (30–50% 1RM) HL (70% 1RM)	12 12	Leg curl Leg extension Leg press	12 wk; 2 days/wk	MRI	LL-BFR: 7% HL: 6%	No significant between-group differences
Libardi et al. [82]	Older adults (> 60 y)	LL-BFR (20–30% 1RM) HL (70–80% 1RM)	10 8	Leg press	12 wk; 2 days/wk	MRI	LL-BFR: 8% HL: 7%	No significant between-group differences
Thiebaud et al. [85]	Older women (61 ± 5 y)	LL-BFR (10–30% 1RM) HL (70–90% 1RM)	6 8	Seated chest press Seated row Seated shoulder press	8 wk; 3 days/wk	Ultrasound Biceps brachii Triceps brachii Deltoid Pectoralis major DEXA Arm bone-free LBM	LL-BFR: 3–17% HL: − 5 to 7%	No significant between-group differences
Vechin et al. [86]	Older adults (59–71 y)	LL-BFR (20–30% 1RM) HL (70–80% 1RM)	8 8	Leg press	12 wk; 2 days/wk	MRI	LL-BFR: 6% HL: 7%	Similar increases in both groups
Yasuda et al. [33]	Older women (61–86 y)	LL-BFR (35–45% 1RM) HL (70–90% 1RM)	10 10	Squats Knee extension	12 wk; 2 days/wk	MRI Quadriceps Adductors Gluteus maximus Hamstring	LL-BFR: 7%^a^ HL: 2%^a^	No significant between-group differences except for quadriceps CSA (greater in LL-BFR)

^a^Values are only reported for the quadriceps muscle, since data for other muscle groups were not available

*1RM* one-repetition maximum, *CON* control group, *CSA* cross-sectional area, *DEXA* dual x-ray absorptiometry, *HL* high-load, *LBM* lean body mass, *LL-BFR* low-load blood flow restriction, *MRI* magnetic resonance imaging, *wk* week/s, *y* years

**Table 3:** BFR walking and changes in muscle strength

Study	Subjects	Protocol	*N*	Exercise mode	Duration/frequency	Strength measurement	Percentage increase	Conclusion
Clarkson et al. [66]	Older adults (60–80 y)	BFR walking (4 km/h) CON walking (4 km/h)	10 9	Walking	6 wk; 4 days/wk	30-sec sit-to-stand test	BFR: 28% CON: 8%	Significantly greater strength increases for BFR
Ozaki et al. [87]	Older adults (57–76 y)	BFR walking (45% HRR) CON walking (45% HRR)	13 10	Treadmill walking (20 min)	10 wk; 4 days/wk	Isokinetic knee extension Isokinetic knee flexion	BFR: 9–15% CON: 0–3%	Significantly greater strength increases for BFR except for knee extension
Ozaki et al. [67]	Older women (57–73 y)	BFR walking (45% HRR) CON walking (45% HRR)	10 8	Treadmill walking (20 min)	10 wk; 4 days/wk	Isometric knee extension Isokinetic knee extension 30°/s; 180°/s Isokinetic knee flexion 30°/s; 180°/s	BFR: 3–22% CON: − 4 to 2%	Significantly greater strength increases for BFR except for isometric knee extension

*1RM* one-repetition maximum, *BFR* blood flow restriction, *CON* control group, *HRR* heart rate reserve, *wk* week/s, *y* years

**Table 4:** BFR walking and changes in muscle mass

Study	Subjects	Protocol	*N*	Exercise mode	Duration/frequency	Muscle mass assessment	Percentage increase	Conclusion
Ozaki et al. [87]	Older adults (57–76 y)	BFR walking (45% HRR) CON walking (45% HRR)	13 10	Treadmill walking (20 min)	10 wk, 4 days/wk	MRI	BFR: 3% CON: 0%	Significant greater muscle mass increases for BFR
Ozaki et al. [67]	Older women (57–73 y)	BFR walking (45% HRR) CON walking (45% HRR)	10 8	Treadmill walking (20 min)	10 wk, 4 days/wk	MRI Mid-thigh (CSA) Mid-quadriceps (CSA) Thigh (volume) Quadriceps (volume)	BFR: 3–4% CON: − 2 to 0%	Significant greater muscle mass increases for BFR

*BFR* blood flow restriction, *CON* control group, *CSA* cross-sectional area, *HRR* heart rate reserve, *min* minutes, *MRI* magnetic resonance imaging, *wk* week/s, *y* years

### Risk of Bias

Following the instructions in the Cochrane Handbook for Systematic Reviews of Interventions [26], risk of bias was assessed using six criteria that were individually rated for each study. In this context, selection bias, performance bias, detection bias, as well as attrition and reporting bias were considered by the reviewers (Electronic Supplementary Material Figure S1). Additionally, to assess the evidence of publication bias, funnel plots were visually inspected for each outcome criterion (Electronic Supplementary Material Figures S2–S6).

### Synthesis of Results

Percentage changes [((MEAN_post_ − MEAN_pre_)/MEAN_pre_) × 100] of muscle strength and muscle mass were calculated for each study. In case of multiple assessment methods, the minimum and maximum mean value of each method were reported (Tables 1, 2, 3, 4).

### Statistical Analyses

Statistical analyses were performed using RevMan (Review Manager Version 5.3, The Cochrane Collaboration, 2014). For calculating the standardized mean difference (SMD), the difference in pre- and post-intervention mean and standard deviation values of muscle mass and strength for all groups in each study were used. Since we partially observed considerable between-timepoint differences in SD_pre_ and SD_post_, SD_change_ was defined as SD_change_ = root square [(SD_pre_^2^/N_pre_) + (SD_post_^2^/N_post_)] [27]. A forest plot was created to present the SMD and 95% confidence intervals (CIs) of muscle mass and muscular strength for all respective comparisons. All analyses were conducted using a random effects model to account for measurement variability and heterogeneity among the studies. For each comparison, pooled effects sizes (ES) were calculated. Alpha level was therefore set to *p* < 0.05. Data are reported as mean ± standard deviation.

The assessment of the between-study heterogeneity was verified with the *I*^2^ method, with an *I*^2^ of 0–40% representing a low heterogeneity, 30–60% representing a moderate heterogeneity and 50–90% and 75–100% representing a substantial or considerable heterogeneity, respectively [26].

In total, five meta-analyses were conducted. First of all, the effects of LL-BFR training on muscle mass and strength were compared with HL training and LL training (analyses 1–3). A fourth and fifth comparison were performed to investigate the additional benefit of blood flow restriction in combination with walking exercise. In all analyses, multiple comparisons were included from several studies (e.g. dynamic and isometric strength measurements) in order to increase accuracy and thus generalization of our analyses. This is a common and accepted statistical method for meta-analysis [28].

## Results

### Study Selection

In total, from an initial 2658 studies, 11 were included in this systematic review and meta-analysis. From 18 studies, we assessed the full texts (for full search process see Fig. 1). After checking for eligibility of these articles based on our inclusion and exclusion criteria, we excluded studies that compared LL-BFR training with balance training [29], water-based exercise [30] or a non-training control group [31, 32]. Additionally, after the corresponding author could repeatedly not be contacted, muscle mass values from one study [33] and all outcome measures from another study were excluded from the meta-analyses [5]. Two more studies had to be excluded due to insufficient study quality (PEDro score < 4) [34, 35].

### LL-BFR versus HL

**Fig. 2:**
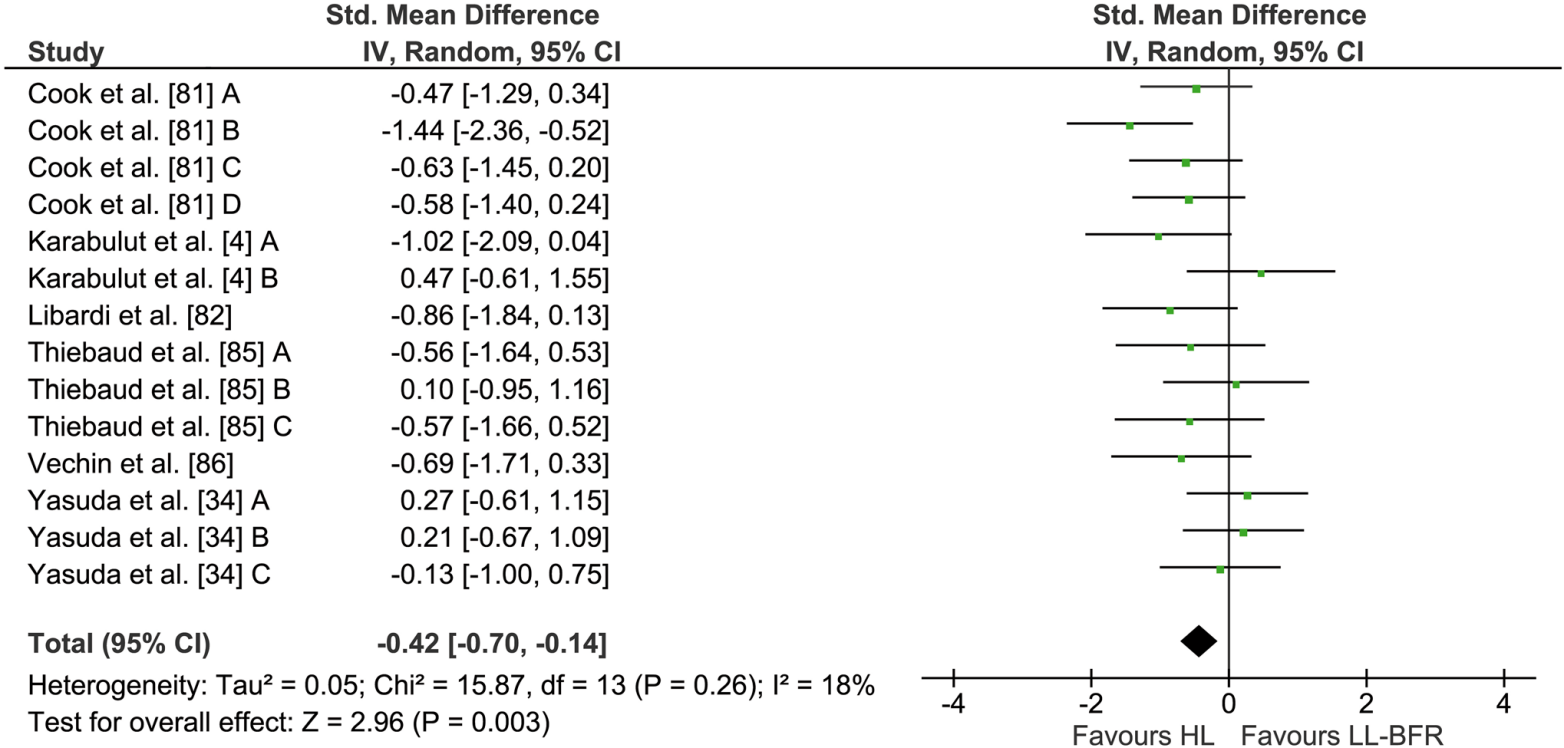
Forest plot demonstrating the effects of LL-BFR versus HL training on muscular strength. Different letters for the same study represent different muscular strength assessment methods. *CI* confidence interval, *HL* high-load, *IV* inverse variance, *LL-BFR* low-load blood flow restriction, *Random* random effects model

Six studies comparing the effects of LL-BFR and HL training on muscle strength were included in the meta-analysis (see Fig. 2). Given that several studies had multiple treatment outcome measures, a total of 14 comparisons were incorporated in the quantitative analysis. Between-group comparisons revealed significantly higher increases in muscle strength following HL (24.0 ± 16.2%) compared with LL-BFR training (14.4 ± 6.3%). The calculation of the meta-analysis showed a significant (*Z* = 2.96, *p* < 0.01) pooled ES of − 0.42 (95% CI − 0.70 to − 0.14) in favour of HL. Heterogeneity was not significant with an *I*^2^ of 18% (*p* = 0.26).

**Fig. 3:**
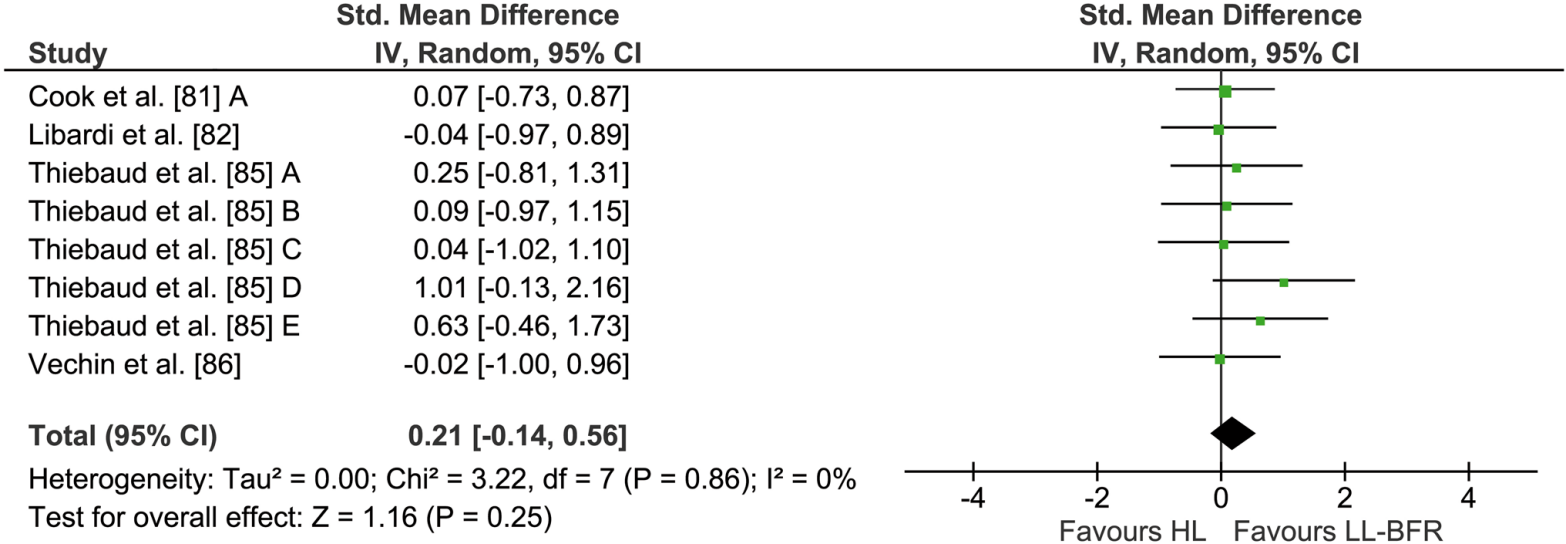
Forest plot demonstrating the effects of LL-BFR versus HL training on muscle mass. Different letters for the same study represent different assessment methods for muscle mass. *CI* confidence interval, *HL* high-load, *IV* inverse variance, *LL-BFR* low-load blood flow restriction, *Random* random effects model

Four studies with eight outcome measures investigated the effects of long-term LL-BFR and HL training on muscle mass (see Fig. 3). Averaged percentage increases of muscle mass were 6.2 ± 5.1% and 4.2 ± 4.2% in the LL-BFR and HL groups, respectively. The weighted average ES was 0.21 (95% CI − 0.14 to 0.56) in favour of LL-BFR training. However, this effect did not reach statistical significance (*Z* = 1.16, *p* = 0.25). The calculation of *I*^2^ showed a heterogeneity of 0% (*p* = 0.86).

### LL-BFR Versus LL

**Fig. 4:**
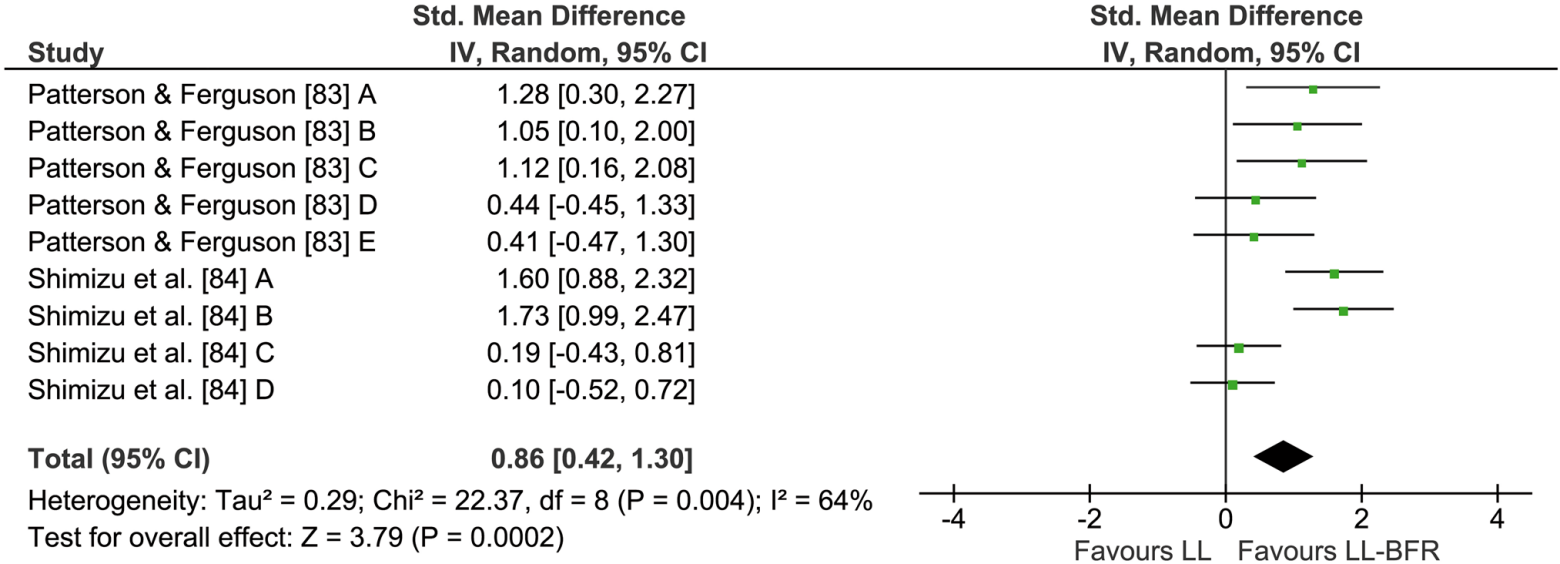
Forest plot demonstrating the effects of LL-BFR versus LL training on muscular strength. Different letters for the same study represent different muscular strength assessment methods. *CI* confidence interval, *IV* inverse variance, *LL* low-load, *LL-BFR* low-load blood flow restriction, *Random* random effects model

A total of two studies and nine comparisons measuring muscular strength following LL-BFR and LL training were included in this meta-analysis (see Fig. 4). Both studies used repetition matched protocols. Across all comparisons, LL-BFR training had an average percentage increase of 12.3 ± 4.1% in muscle strength, compared with LL with 2.5 ± 2.7%. Quantitative analyses demonstrated significantly greater strength increases with LL-BFR compared with LL (*Z* = 3.79, *p* < 0.001). The pooled ES was 0.86 (95% CI 0.42–1.30). However, heterogeneity was considerably higher for this meta-analysis with *I*^2^ = 64% (*p* < 0.01).

No study was identified comparing the effects of LL-BFR and LL on muscle mass.

### BFR and Walking

**Fig. 5:**
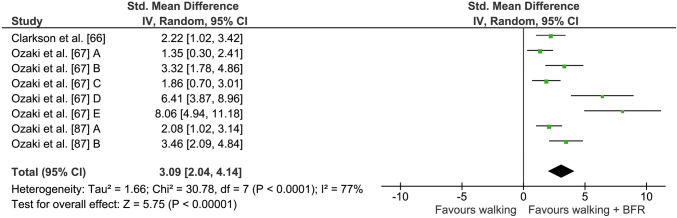
Forest plot demonstrating the effects of walking + BFR versus normal walking on muscular strength. Different letters for the same study represent different muscular strength assessment methods. *BFR* blood flow restriction, *CI* confidence interval, *IV* inverse variance, *Random* random effects model

Three studies (eight comparisons) assessed muscle strength changes following long-term BFR walking and walking with normal blood flow (see Fig. 5). Studies that combined walking with and without BFR showed percentage changes of 13.3 ± 8.5% and 0.4 ± 3.9% in muscular strength, respectively. Calculation of the meta-analysis revealed significantly greater strength increases (*Z* = 5.75, *p* < 0.001) when walking was performed with partial vascular occlusion. The weighted average ES was 3.09 (95% CI 2.04–4.14). *I*^2^ for this analysis was 77% and demonstrated a high heterogeneity (*p* < 0.001).

**Fig. 6:**
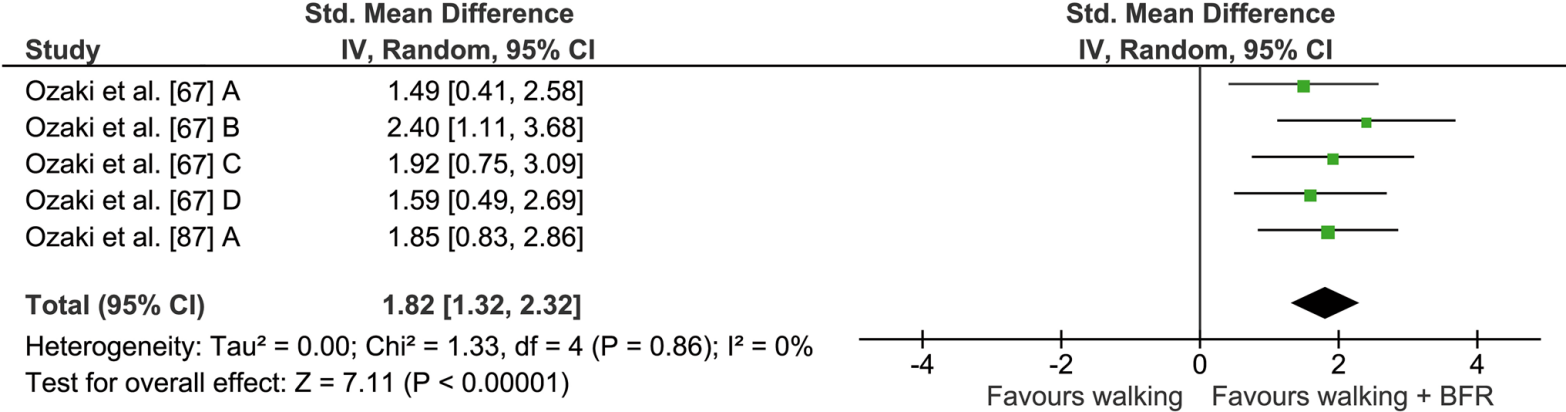
Forest plot demonstrating the effects of walking + BFR versus normal walking on muscle mass. Different letters for the same study represent different muscle mass assessment methods. *BFR* blood flow restriction, *CI* confidence interval, *IV* inverse variance, *Random* random effects model

In order to compare the effects of walking with and without BFR on muscle mass, two studies with a total of seven comparisons were included in the quantitative analysis (see Fig. 6). Mean muscle mass percentage gain was 3.0 ± 0.4% for the BFR + walking group, with mean percentage changes of − 0.7 ± 0.7% in walking with normal blood flow. Statistical examination revealed a significantly higher increase in muscle mass following BFR compared with normal walking (*Z* = 7.11, *p* < 0.001). The average ES and *I*^2^ were 1.82 (95% CI 1.32–2.32) and 0% (*p* = 0.86), respectively.

## Discussion

The main objective of the present systematic review and meta-analysis was to assess the effects of LL-BFR training on muscle mass and strength in older adults, compared with conventional HL and LL training. In an additional analysis, we sought to provide insights into the beneficial effect of BFR combined with walking as this has particular implications for older individuals at risk of mobility limitations.

While our analyses demonstrate that HL and LL-BFR training produce similar increases in muscle mass in older cohorts, adaptations in muscular strength were smaller following LL-BFR training compared with those typically seen after HL training. However, the application of an external tourniquet seems to facilitate significantly greater responses in muscular strength compared with LL training alone. Due to insufficient data availability, no conclusion can be drawn about the effects of LL-BFR training on muscle mass compared with LL training alone. Interestingly, even during intensities as low as walking, BFR enhances strength and muscle mass adaptations in older subjects compared with normal walking.

### LL-BFR Versus HL Resistance Training

Our results suggest that LL-BFR training is equally effective in increasing muscle mass but seems to be inferior in eliciting muscle strength responses compared with a common HL resistance training programme in older subjects. These findings are in line with a previously published meta-analysis by Lixandrao et al. [6], which investigated the effects of LL-BFR training and HL training in a mixed-age population.

Even though mechanical tension produced by LL-BFR training is assumed to be much lower than during HL training, our results indicate that gains in muscle mass were not different between these training protocols in older subjects. One plausible mechanism that has been reported to be as important as mechanical tension for the promotion of muscle mass is metabolic stress [36]. While one study demonstrated an augmented lactate concentration following LL-BFR compared with HL resistance training for older men [5], others showed inconsistent results [37]. Moreover, several studies reported that the intramuscular hypoxic environment and metabolic stress influence the fatigability of the muscle fibres and thus induce a progressive recruitment of motor units during training [38, 39]. In addition to metabolic accumulation, the effects of LL-BFR on muscular hypertrophy have been suggested to be mediated by an increased mechanotransduction [40] and hormonal response [41], an acute production of reactive oxygen species [42] or cell swelling [36, 43]. However, current research on this topic is sparse and studies investigating potential mechanisms are mostly performed with younger populations [39, 44, 45] or do not compare LL-BFR and HL resistance training [39, 40, 46]. Thus, any definite conclusions at this time would be premature.

The inferiority of LL-BFR resistance training in increasing muscular strength compared with traditional HL programmes could be linked to an insufficient neural drive during exercising with low loads. Studies investigating this aspect used surface electromyography (sEMG) or twitch interpolation to estimate changes in voluntary muscle activation during exercise. It was reported that EMG parameters (e.g. amplitude or integrated EMG) were greater following HL training than following LL-BFR training [47–49]. Kubo et al. [49], for example, showed that the activation levels of the quadriceps muscle assessed by sEMG and twitch interpolation significantly increased by 20.5% and 3.2%, respectively, following 12-week HL training, with no significant changes in the LL-BFR group. However, these results must be interpreted with caution since a higher EMG amplitude might not necessarily represent a higher motor unit recruitment. Often the phenomenon of motor unit cycling, which refers to the fact that motor units can be temporarily de-recruited for the purpose of reducing fatigue [50–52], is ignored by researchers. Moreover, these studies were conducted in young and healthy subjects [48, 49] and may not necessarily be transferred to older populations. However, the findings in these cohorts provide insights into how the observed results might be explained in older individuals.

### LL-BFR Versus LL Resistance Training

Our finding that the addition of BFR to LL resistance training enhances muscle strength supports the results of a previous meta-analysis from Slysz et al. [1] that was conducted in mixed-aged populations. Functional adaptations in strength are generally believed to be mediated by neural (e.g. increased muscle activation) and/or structural factors (e.g. muscular hypertrophy) [53]. Evidence on this topic suggests that the application of a cuff during LL training is associated with a reduction in oxygen availability and high metabolite accumulation, thereby leading to significantly increased fast-twitch fibre recruitment [38, 39, 54]. However, studies with protocols to volitional exhaustion reported a similar muscle activation in both LL-BFR and LL groups [46, 48]. This supports the notion that LL alone can also elicit high levels of muscle activity (as assessed with sEMG) if the exercise task is performed in an all-out manner [46]. Accordingly, long-term intervention studies confirm that free-flow LL training performed to fatigue induces equal muscular hypertrophy compared with the same training with BFR [55]. Translating this to older individuals, performing resistance exercise to failure could increase the incidence of overtraining or musculoskeletal injuries compared with young individuals [56]. Therefore, the prescription of LL-BFR resistance training could be beneficial in these populations.

Besides neural changes with LL-BFR training, there seem to be structural changes when combining LL training with BFR in older people. Although two studies investigated the effects of LL-BFR in older individuals, both could not be included in the present meta-analysis due to insufficient study quality (PEDro < 4) [34] or unavailable raw data [5]. However, their findings point towards a significantly greater increase of muscle mass in the LL-BFR group compared with the LL group. Studies investigating the increase of muscle mass following LL-BFR in young individuals [57, 58] or athletes [59] confirm these results and show that LL-BFR maximizes the effects of LL training on muscle mass. These results, however, do not permit reliable statements for older populations.

Previous short-term studies provided evidence that the hypertrophic response is upregulated with partial vascular occlusion in older subjects. Fry et al. [40] investigated the effects of BFR training on stimulating mammalian target of rapamycin (mTOR) and muscle protein synthesis (MPS). Their results demonstrate that LL-BFR enhances mTOR signalling and MPS. Additionally, Fry and colleagues [40] observed a significant 9-fold growth hormone (GH) increase in the LL-BFR group compared with the control group. These findings are in accordance with other studies [39, 60], but have to be cautiously interpreted with regard to muscular hypertrophy, since muscle growth can occur even in the absence of key anabolic hormones such as insulin-like growth factor 1 or GH [61–64].

### BFR Walking Versus Normal Walking

Although long-term walking training has been shown to increase muscle thickness and strength in the elderly [65], the present meta-analysis revealed that the combination of walking with BFR has significant additional benefits towards these outcomes. The percentage changes in muscular strength (+ 13.3%) are comparable to what is seen after LL resistance training (+ 12.3%). Changes in muscle mass are small but still significant (+ 3.0%). Previous studies also report an increase in physical function [66], but not in aerobic capacity (estimated by peak oxygen uptake, VO_2peak_) [32, 67].

Interestingly, a recent meta-analysis by Slysz et al. showed that the strength adaptations occur in an intensity-dependent manner, with higher walking intensities (> 70 m/min) eliciting greater strength increases compared with lower intensities (< 70 m/min) [1]. Besides its positive effects on muscle mass and strength, walking combined with BFR has also been shown to improve venous compliance in untrained elderly subjects [68].

### Practical Implications

Numerous studies have shown that the age-related loss of muscle strength is associated with a decrease in postural control [69] leading to a higher risk of falls [70] and mortality [71]. A frequently occurring simultaneous loss in muscle mass can contribute to the development of cardiometabolic diseases in the elderly [72]. This highlights the need for adequate interventions to counteract these phenomena in older age. To maximize the span of effective functioning for people with sarcopenia, Cruz-Jentoft et al. [73] suggest the use of multidimensional approaches combining physical exercise and nutritional interventions. However, current exercise guidelines for older people [74] are often difficult to implement due to contraindications to high training loads.

The fact that even low-workload walking exercise is able to facilitate such changes is of particular importance for older populations with limited functional capacity or mobility [11, 75]. Maintaining fitness and an active lifestyle could thus help to postpone the crossing of the threshold for independence [76]. Apart from improvements on the muscular level, LL-BFR training has also been shown to positively influence bone metabolism and hence may be applicable in the prevention and treatment of bone diseases such as osteoporosis [77].

Given these musculoskeletal adaptations, LL-BFR training may be particularly recommended for older populations with contraindications regarding high training loads. For healthy individuals without contraindications, LL-BFR training may be prescribed in combination with HL training in order to aim for optimal muscular strength responses. From a practical standpoint, the data from our meta-analysis might help practitioners and therapists in geriatrics and rehabilitation to increase clients’ functional capacity and maintain quality of life. In this regard, taking a thorough cardiovascular disease history from each individual is important to avoid adverse events, particularly since most risk factors have not been thoroughly investigated in older people. Kacin et al. [78] have developed a clinical screening tool for determining risk when prescribing BFR training programmes. These authors recommend a comprehensive assessment of personal, medical, social and family histories.

### Limitations and Strengths

Regarding the interpretation of our results, there are some limitations in the present meta-analysis that should be mentioned. Although the field of BFR training is a frequently discussed topic in scientific research [3, 7, 21, 77], the number of studies investigating the effects of LL-BFR in older adults is still sparse. The limited number of included studies (*N* = 11) is not least attributable to the fact that we intentionally chose strict inclusion criteria in terms of study quality (PEDro > 4). It must also be noted that the study quality of the majority of included studies (10/11) was only rated as moderate (PEDro = 4). One main factor for potential bias and thus restricted study quality in all studies was the lack of subject blinding. While we are aware that it is not always feasible in BFR training interventions, future investigations should aim to choose different training locations in order to reduce performance bias. In addition, a large heterogeneity was found across studies for the comparisons of BFR with LL (*I*^2^ = 64) and walking (*I*^2^ = 77%) in muscular strength assessments. This large variability might result from differences in training protocols (i.e. training durations from 4 to 10 weeks), sample sizes, trained limbs (i.e. lower vs upper extremity) and strength assessments (dynamic 1RM vs isometric vs isokinetic testing). Furthermore, considering multiple outcomes from the same study in one meta-analysis could also partially have an impact on the homogeneity of the results.

## Conclusion

The present systematic review and meta-analysis provides novel insights into the effect of LL-BFR training compared with training modalities that are currently used for counteracting the age-related decline in muscle mass and function. Our results indicate that the application of BFR to LL training and walking exercise positively influences muscular adaptations compared with each exercise under normal blood-flow conditions. In comparison with HL training, LL-BFR elicits lower strength increases.

Although the research on this topic is limited, our data provide first evidence for practitioners and physicians that are confronted with individuals that cannot tolerate near-maximum loads but are in need of adequate therapy. Although previous surveys and reviews report an acceptable level of safety for LL-BFR for mixed age populations [79, 80], we recommend a thorough screening and physical examination of all trainees before commencing this training regimen. Although it was beyond the scope of this review, future studies need to examine potential moderators (e.g. cuff pressure, sex, volume or frequency) that might affect adaptations of muscle mass and strength in older adults. Additionally, we want to draw attention to the lack of high-quality studies comparing the effects of LL-BFR and LL on muscle mass.

### Data Availability Statement

The datasets generated and analysed during the current systematic review and meta-analysis are available from the corresponding author on reasonable request.

## Electronic supplementary material

Below is the link to the electronic supplementary material.

Supplementary material 1 (DOCX 112 kb)
