# Supplementary material for: Effects of Blood Flow Restriction Training on Muscular Strength and Hypertrophy in Older Individuals: A Systematic Review and Meta-Analysis
Source: Sports Med. 2018 Oct 10;49(1):95–108. doi: 10.1007/s40279-018-0994-1 (PMC6349784; doi:10.1007/s40279-018-0994-1)
Supplement: Supplementary file 1 — Supplementary material 1 (DOCX 112 kb) [file 40279_2018_994_MOESM1_ESM.docx]

**Effects of Blood Flow Restriction Training on Muscular Strength and Hypertrophy in the Elderly: A Systematic Review and Meta-Analysis**

**Journal Name:** Sports Medicine

Christoph Centner^1^, Patrick Wiegel^1,2^, Albert Gollhofer^1^, Daniel König^1^

^1^ *Department of Sport and Sport Science, University of Freiburg, Germany*

^2^ *Bernstein Center Freiburg, University of Freiburg, Germany*

**Corresponding Author:** Christoph Centner, M.Sc.

**E-Mail:** christoph.centner@sport.uni-freiburg.de

**Ph:** +4976120354087

**Electronic Supplemental Material**

**PEDro Scale**

**Electronic Supplemental Material Table S1** Physiotherapy Evidence Database (PEDro) scores for each of the 11 included studies. 1 = criterion is satisfied; 0 = criterion not satisfied

|  | **Study** **quality** **criterion** | | | | | | | | | | | | | | | | | | | | | |  |  |
| --- | --- | --- | --- | --- | --- | --- | --- | --- | --- | --- | --- | --- | --- | --- | --- | --- | --- | --- | --- | --- | --- | --- | --- | --- |
| **Study** | 2 | | 3 | | 4 | | 5 | | 6 | | 7 | | 8 | | 9 | | 10 | | 11 | **Total** | | |  |  |
| **Clarkson et al. (2017)** | 1 | | 0 | | 1 | | 0 | | 0 | | 0 | | 0 | | 0 | | 1 | | 1 | **4** | |  |  |  |
| **Cook et al. (2017)** | 1 | | 0 | | 1 | | 0 | | 0 | | 1 | | 1 | | 0 | | 1 | | 1 | **6** | |  |  |  |
| **Karabulut et al. (2010)** | 1 | | 0 | | 0 | | 0 | | 0 | | 0 | | 1 | | 0 | | 1 | | 1 | **4** | |  |  |  |
| **Libardi et al. (2015)** | 1 | | 0 | | 1 | | 0 | | 0 | | 0 | | 0 | | 0 | | 1 | | 1 | **4** | |  |  |  |
| **Ozaki et al. (2011a)** | 0 | | 0 | | 1 | | 0 | | 0 | | 1 | | 0 | | 0 | | 1 | | 1 | **4** | |  |  |  |
| **Ozaki et al. (2011b)** | 1 | | 0 | | 1 | | 0 | | 0 | | 0 | | 1 | | 1 | | 1 | | 1 | **4** | |  |  |  |
| **Patterson et al. (2010)** | 0 | | 0 | | 1 | | 0 | | 0 | | 0 | | 1 | | 1 | | 1 | | 1 | **5** | |  |  |  |
| **Shimizu et al. (2016)** | 1 | | 0 | | 1 | | 0 | | 0 | | 0 | | 0 | | 0 | | 1 | | 1 | **4** | |  |  |  |
| **Thiebaud et al. (2013)** | 0 | | 0 | | 1 | | 0 | | 0 | | 0 | | 1 | | 0 | | 1 | | 1 | **4** | |  |  |  |
| **Vechin et al. (2015)** | 1 | | 0 | | 1 | | 0 | | 0 | | 0 | | 0 | | 0 | | 1 | | 1 | **4** | |  |  |  |
| **Yasuda et al. (2016)** | 1 | | 0 | | 1 | | 0 | | 0 | | 0 | | 0 | | 0 | | 1 | | 1 | **4** | |  |  |  |
|  |  |  | |  | |  | |  | |  | |  | |  | |  | |  | | |  | | |  |

**Risk of Bias**


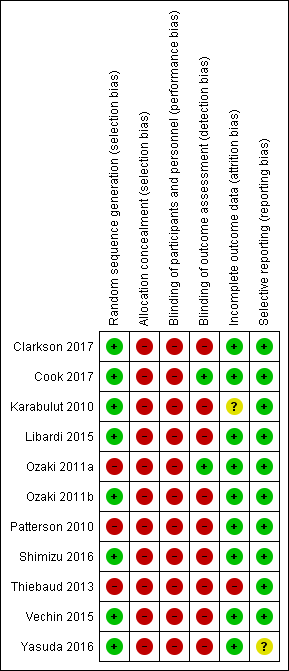


**Electronic Supplemental Material Figure S1** Risk of bias for each study

**Funnel plots**

**
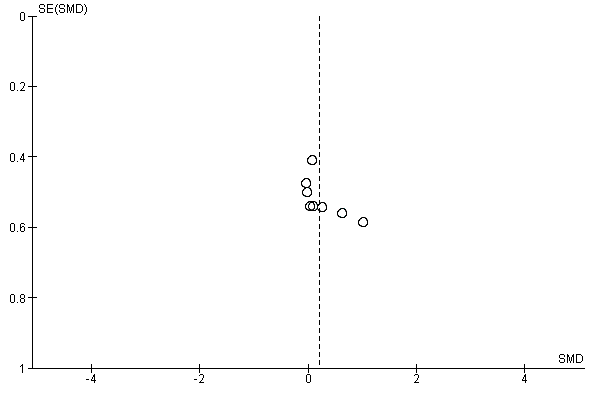
**

**Electronic Supplemental Material Figure S2** Funnel plot of the comparison between the effects of low-load BFR and high-load training on muscle mass. SE (SMD) = standard error of SMD; SMD = standardized mean difference


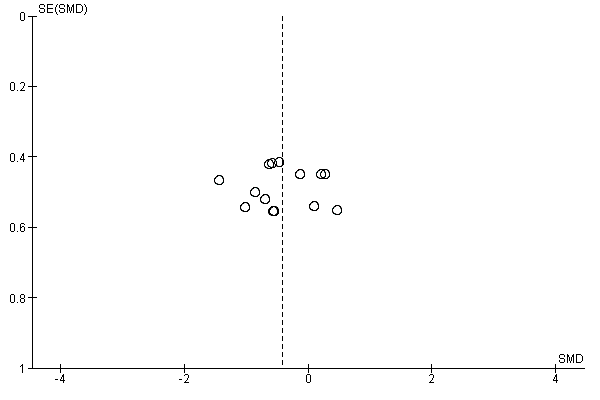


**Electronic Supplemental Material Figure S3** Funnel plot of the comparison between the effects of low-load BFR and high-load training on muscular strength. SE (SMD) = standard error of SMD; SMD = standardized mean difference


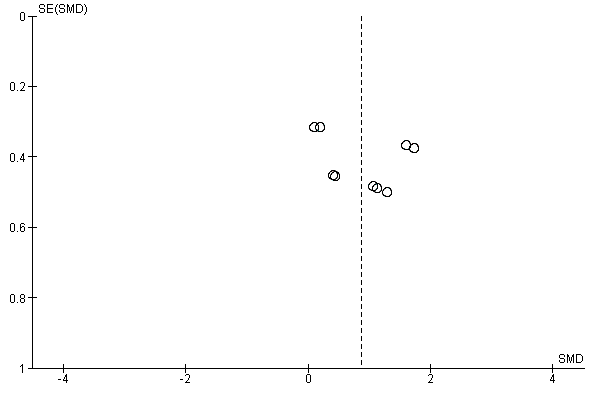


**Electronic Supplemental Material Figure S4** Funnel plot of the comparison between the effects of low-load BFR training and low-load training on muscular strength. SE (SMD) = standard error of SMD; SMD = standardized mean difference


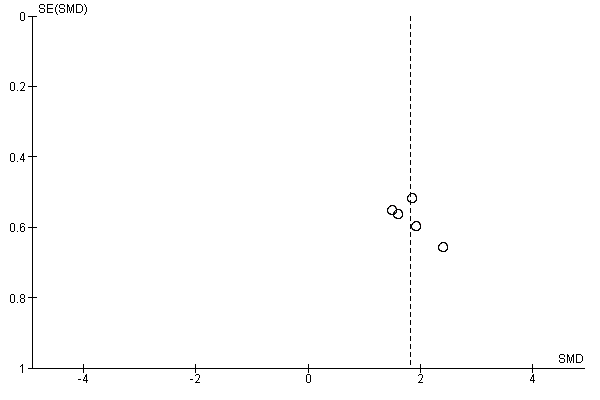


**Electronic Supplemental Material Figure S5** Funnel plot of the comparison between the effects of walking with blood flow restriction and normal walking on muscle mass. SE (SMD) = standard error of SMD; SMD = standardized mean difference


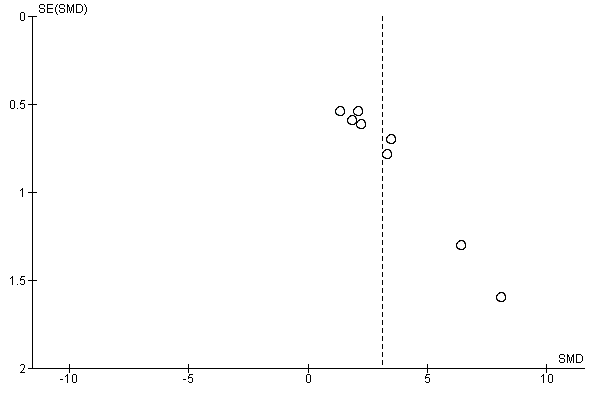


**Electronic Supplemental Material Figure S6** Funnel plot of the comparison between the effects of walking with blood flow restriction and normal walking on muscular strength. SE (SMD) = standard error of SMD; SMD = standardized mean difference

Clarkson, M. J., Conway, L., & Warmington, S. A. (2017). Blood flow restriction walking and physical function in older adults: A randomized control trial. *J Sci Med Sport, 20*(12), 1041-1046. doi:10.1016/j.jsams.2017.04.012

Cook, S. B., LaRoche, D. P., Villa, M. R., Barile, H., & Manini, T. M. (2017). Blood flow restricted resistance training in older adults at risk of mobility limitations. *Exp Gerontol, 99*, 138-145. doi:10.1016/j.exger.2017.10.004

Karabulut, M., Abe, T., Sato, Y., & Bemben, M. G. (2010). The effects of low-intensity resistance training with vascular restriction on leg muscle strength in older men. *Eur J Appl Physiol, 108*(1), 147-155. doi:10.1007/s00421-009-1204-5

Libardi, C. A., Chacon-Mikahil, M. P., Cavaglieri, C. R., Tricoli, V., Roschel, H., Vechin, F. C., . . . Ugrinowitsch, C. (2015). Effect of concurrent training with blood flow restriction in the elderly. *Int J Sports Med, 36*(5), 395-399. doi:10.1055/s-0034-1390496

Ozaki, H., Miyachi, M., Nakajima, T., & Abe, T. (2011a). Effects of 10 weeks walk training with leg blood flow reduction on carotid arterial compliance and muscle size in the elderly adults. *Angiology, 62*(1), 81-86. doi:10.1177/0003319710375942

Ozaki, H., Sakamaki, M., Yasuda, T., Fujita, S., Ogasawara, R., Sugaya, M., . . . Abe, T. (2011b). Increases in thigh muscle volume and strength by walk training with leg blood flow reduction in older participants. *J Gerontol A Biol Sci Med Sci, 66*(3), 257-263. doi:10.1093/gerona/glq182

Patterson, S. D., & Ferguson, R. A. (2010). Increase in calf post-occlusive blood flow and strength following short-term resistance exercise training with blood flow restriction in young women. *Eur J Appl Physiol, 108*(5), 1025-1033. doi:10.1007/s00421-009-1309-x

Shimizu, R., Hotta, K., Yamamoto, S., Matsumoto, T., Kamiya, K., Kato, M., . . . Masuda, T. (2016). Low-intensity resistance training with blood flow restriction improves vascular endothelial function and peripheral blood circulation in healthy elderly people. *Eur J Appl Physiol, 116*(4), 749-757. doi:10.1007/s00421-016-3328-8

Thiebaud, R. S., Loenneke, J. P., Fahs, C. A., Rossow, L. M., Kim, D., Abe, T., . . . Bemben, M. G. (2013). The effects of elastic band resistance training combined with blood flow restriction on strength, total bone-free lean body mass and muscle thickness in postmenopausal women. *Clin Physiol Funct Imaging, 33*(5), 344-352. doi:10.1111/cpf.12033

Yasuda, T., Fukumura, K., Tomaru, T., & Nakajima, T. (2016). Thigh muscle size and vascular function after blood flow-restricted elastic band training in older women. *Oncotarget, 7*(23), 33595-33607. doi:10.18632/oncotarget.9564
